# P-1824. AI-Powered Differential Diagnosis: Distinguishing MIS-C from Kawasaki Disease

**DOI:** 10.1093/ofid/ofae631.1987

**Published:** 2025-01-29

**Authors:** Aditya Basu, Surupa Basu

**Affiliations:** Triomics Healthcare, Kolkata, West Bengal, India; Institute of Child Health, Kolkata, Kolkata, West Bengal, India

## Abstract

**Background:**

Distinguishing Multisystem Inflammatory Syndrome in Children from COVID-19 (MIS-C) from Kawasaki Disease (KD) can be challenging due to several overlapping clinical presentations. Clinical management of these conditions can vary. Hence, timely and accurate diagnosis is essential. We evaluated the potential of laboratory findings in the differentiation using machine learning (ML) models.

**Methods:**

Patient demographics (Age, Sex) and laboratory data were compared between MIS-C and KD. Simultaneous CBC and routine biochemistry tests were evaluated. ML models were created using PyTorch, scikit-learn, Pandas, NumPy, and Matplotlib libraries. Specifically, Naive Bayes Classifier, KNN, Logistic Regression, SVC, Random Forest Classifier, XGBoost, GBM and LightGBM models were created and the model performances of the classifiers were evaluated with accuracy percentages. The confusion matrix of every learning model was also generated. K-fold CrossValidation was also performed to evaluate the predictive models. ROC and feature importance analysis were also performed. Identification of the disease as MIS-C or KD based on both regressive (Eg: Total Protein) and binary features (Eg: Sex) is posed as a binary classification ML problem. Train and test datasets were created using a couple of train-test splits (0.2 and 0.25).

**Results:**

209 paediatric patients with MIS-C (n=140), and KD (n=69) were included.

• The accuracy performances of the models in the test set were determined as 67.8, 73.8, 75.9, 76.8, 79.9, 80.4, 81.0, 81.1%, respectively.

• The area under curve (AUC) values of the LightGBM, GBM, XGBoost, were determined as 79.1, 79.5 and 78%, respectively.

• Permutation importance analysis of the LightGBM model showed that elevated platelets, neutrophil count, SGPT, WBC levels, Creatinine, and NT ProBNP had influenced the model’s explainability.

**Conclusion:**

Although four years have passed since the onset of COVID-19, the early diagnosis of MIS-C is still crucial. In addition to clinical findings, laboratory tests may contribute to the fight against the disease by providing early diagnosis of MIS-C and KD. Explainable ML models can help determine the appropriate markers for this purpose.

**Disclosures:**

**Aditya Basu, Bachelor of Technology**, Triomics Healthcare: Stocks/Bonds (Private Company)

